# Fault-Tolerant Control of AGVs via Deep Feature Enhancement and Multi-Source Verification in Complex Industrial Environments

**DOI:** 10.3390/s26113428

**Published:** 2026-05-28

**Authors:** Yazhou Zhou, Shanshan Peng, Yun Wang, Nan Zhou, Fei Shan

**Affiliations:** 1 School of Mechanical Engineering, Jiangsu University, 301 Xuefu Road, Zhenjiang 212013, China; 2112403005@stmail.ujs.edu.cn; 2 Suzhou Yuanzi Intelligent Technology Co., Ltd., 381 Suzhou Avenue, Suzhou 215000, China; pss@yuanzirobot.com.cn (S.P.); zhounan@just.edu.cn (N.Z.); f.shan@just.edu.cn (F.S.); 3 College of Oceanography, Jiangsu University of Science and Technology, 2 Mengxi Road, Zhenjiang 212134, China

**Keywords:** automated guided vehicle, YOLOv8, anomaly detection, adaptive decision making, robust sensing, industrial material handling

## Abstract

To address the issue of 2D laser-guided automated guided vehicles (AGVs) in industrial intelligent material handling scenarios being susceptible to interference from changes in lighting and complex obstacles, leading to abnormal positioning and mapping and frequent false stops, this paper designs a lightweight, multi-dimensional perception and anti-false-stop YOLOv8 anomaly recognition network, achieving accurate identification of various interferences in complex environments. An adaptive decision-making fault-tolerant control algorithm is proposed, introducing a temporal logic verification and dynamic threshold adjustment mechanism to achieve real-time dynamic switching of obstacle avoidance levels, ensuring efficient coordination between perception decision-making and control execution. An AGV anomaly detection sample set suitable for complex industrial scenarios is constructed, providing reliable data support for model optimization and accuracy evaluation. Finally, real-world deployment verification in a real electronics factory environment shows that this method reduces the vehicle false-stop rate and improves task handling efficiency. This research effectively solves the robust perception problem of AGVs in complex industrial environments and has significant engineering application value.

## 1. Introduction

Against the backdrop of deep transformation in intelligent manufacturing, automated guided vehicles (AGVs), as the core carrier of flexible logistics, directly determine the operational efficiency of factories based on the robustness of their perception [1,2]. However, in industrial scenarios with complex lighting and dense equipment, such as electronics factories, mainstream laser guidance solutions face severe challenges: due to their high dependence on geometric features, they are prone to echo voids or “false wall” effects when encountering specular reflection, drastic changes in lighting, or fence structures [3]. This ambiguity at the perception level leads to frequent false stops, becoming a bottleneck restricting the efficient operation of the system [4].

To address the aforementioned limitations, researchers have conducted extensive research in the field of multi-sensor fusion. Yuan et al. [5] proposed a multi-source fusion positioning model integrating odometer, IMU, lidar and ultra-wideband (UWB) to address the problem of cumulative errors that easily occur between two-dimensional lidar and inertial navigation systems indoors. They used the unscented Kalman filter (UKF) algorithm to achieve deep coupling of multi-dimensional data. Song et al. [6] developed a hybrid sensing pipeline, which used a fully convolutional neural network (FCN) to perform semantic segmentation on camera and lidar data and combined it with Kalman filtering to achieve asynchronous sensor state tracking, verifying the effectiveness of cross-modal feature complementarity in improving sensing reliability. Zhou et al. [7] systematically reviewed various navigation methods and pointed out that multi-sensor information fusion (MSIF) is an inevitable trend to overcome the navigation stability of AGVs in complex industrial environments. They also discussed the balance between efficiency and real-time performance in heterogeneous data processing.

However, there are still three technical gaps in practical engineering applications: First, insufficient dataset support: Although the KITTI dataset established by Geiger et al. [8] laid the foundation for outdoor perception, it focuses on conventional road environments and lacks a special sample library for industrial sites (such as mirror images and light and shadow in narrow alleys), which limits the generalization ability of the model under extreme conditions. Second, insufficient decoupling between physical and semantic features: Although Yang et al. [9] and Qian et al. [10] optimized the recognition accuracy of YOLOv5 through attention mechanism and gated convolution, such models still perform visual tasks in isolation. Due to the lack of a consistency constraint mechanism for deep coupling between laser physical echo and visual semantics, the system is difficult to eliminate “pseudo-obstacle” interference from a physical nature. Finally, the lack of coordination between perception uncertainty and control execution. Existing systems generally lack fault tolerance processing for perception uncertainty. Hu et al. [11] pointed out that the inference variability of perception models in complex environments leads to a significant increase in decision-making risks, while traditional logic tends to be overly conservative when dealing with perceptual uncertainty, which easily triggers unnecessary emergency braking. As Li et al. [12] stated, rigid control without a multi-stage deceleration strategy can lead to a conflict between the reference path and the execution capability; furthermore, Han et al. [13] pointed out that such an architecture lacking robust fault tolerance mechanism can exacerbate mechanical wear and lead to path tracking errors.

To address the aforementioned challenges, this paper proposes a robust perception framework for AGVs based on multi-source information logical constraints and adaptive decision-making. The main contributions are summarized as follows:

- A comprehensive AGV anomaly detection dataset tailored for complex industrial scenarios is constructed. It includes real-world samples such as high-dynamic obstacles (High), ground dynamic interference (Ground), geometric structural ambiguity (Fence), mirror virtual image interference (Mirror), and severe lighting/shadow interference (Light), providing a solid data foundation for model optimization and accuracy evaluation.
- Building upon the stability of YOLOv8, a lightweight multi-dimensional perception and anti-false-stop recognition network is developed. By deeply integrating visual semantics, LiDAR physical characteristics, and temporal motion constraints, the network achieves precise identification of various environmental interferences, significantly enhancing the operational continuity of AGVs.
- An adaptive decision-making and fault-tolerant control algorithm is proposed. To handle discontinuities and uncertainties in the perceived environment, temporal logic verification and dynamic threshold adjustment mechanisms are introduced, enabling the system to switch obstacle avoidance levels in real time based on target confidence. This algorithm effectively prevents frequent emergency braking under boundary conditions, ensuring efficient coordination between perception, decision-making, and control execution in complex dynamic scenarios.

The remainder of this paper is organized as follows: Section 2 introduces the overall system framework and the improved detection network, focusing on the design principles of the AG-FPN module, DHA module, multi-dimensional pseudo-obstacle discrimination model, and fault-tolerant control strategy. Section 3 details the experimental validation and results analysis, covering dataset construction, ablation studies, and real-world vehicle testing. Finally, Section 4 concludes the paper and discusses future research directions.

## 2. System Overall Framework

### 2.1. Framework Overview and Operating Logic

**Figure 1:**
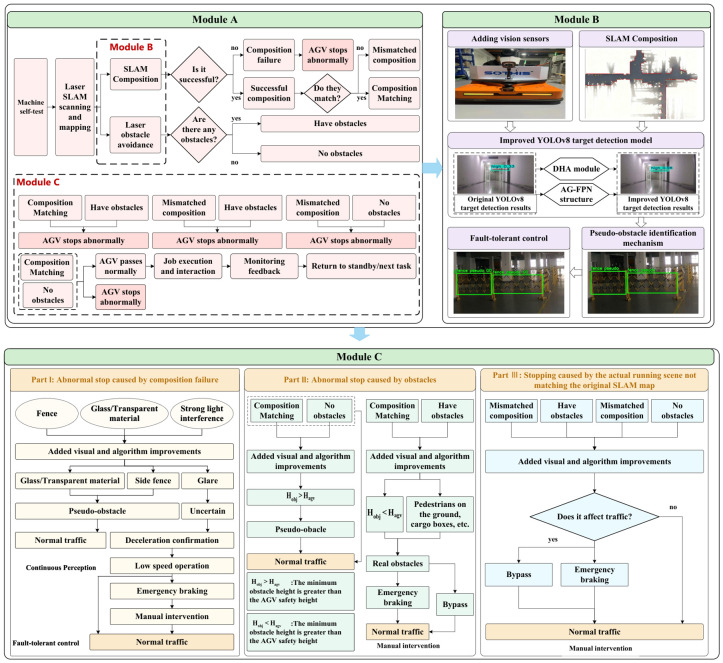
Architecture of the proposed collaborative sensing and control framework. The system consists of three collaborative stages: (**A**) **Foundational Navigation Stage**: Performs SLAM-based localization and triggers vision-assisted perception upon anomalies; (**B**) **Vision Enhancement Stage**: Utilizes improved YOLOv8 (with AG-FPN and DHA modules) and multi-dimensional consistency verification to filter “pseudo-obstacles”; (**C**) **Adaptive Decision-Making Stage**: Executes hierarchical fault-tolerant control to dynamically adjust avoidance levels based on risk assessment.

The system operation logic proposed in this paper is shown in Figure 1, and is mainly divided into the following three functional stages:

Basic navigation and status monitoring stage (Module A): The vehicle relies on 2D LiDAR to execute the SLAM algorithm to achieve environmental mapping and autonomous localization. The system monitors the raw sensor data and positioning status in real time. Once an anomaly in the LiDAR point cloud or a potential obstacle signal is detected, the visual-assisted perception mode is immediately triggered.

Visual Enhancement and Multidimensional False Obstacle Recognition Stage (Module B): This module is the core recognition layer of the system, designed to eliminate perceptual ambiguity. First, an improved lightweight YOLOv8 algorithm (integrating AG-FPN and DHA modules) is used to extract high-dimensional semantic features, achieving accurate targeting of multi-scale targets in complex industrial environments. Subsequently, the system performs a multi-dimensional consistency verification by mapping visual semantic results into a unified spatial coordinate system, integrating LiDAR echo intensity, geometric height, and temporal states. This mechanism utilizes physical occupancy features to logically calibrate visual semantic outputs, effectively decoupling “pseudo-obstacles” (such as intense specular reflections and phantom images) at the decision level. Consequently, the proposed approach significantly enhances the system’s discriminative capability and robustness against complex environmental interferences.

Adaptive Decision-Making and Hierarchical Fault-Tolerant Control Stage (Module C): This stage is responsible for executing a closed-loop response to the perceptual decision results. Based on a risk assessment model, the system triggers a hierarchical response strategy according to target attributes and position confidence. By dynamically switching between adaptive deceleration, path detour, and emergency braking, the system ensures that the AGV maintains operational continuity under uncertain conditions, effectively reducing unnecessary stoppages.

### 2.2. Improve Detection Network

The detection network based on YOLOv8 designed in this paper mainly consists of the following three parts:

1. **Backbone layer**: Extracts basic features of the AGV operating environment through multi-layer convolution and residual structure [14], including edge, texture and geometric information, to provide multi-level semantic representation for subsequent feature fusion and detection.
2. **Neck layer**: Adopts improved AG-FPN structure and improves the transmission and fusion effect of cross-scale information by reducing computational complexity through efficient feature reuse [15].
3. **Head front end**: Embeds DHA module in the front end of the detection head and improves the feature discrimination and capture ability of the model for abnormal regions and difficult-to-detect targets by parallel fusion of saliency, channel and spatial attention mechanisms. It adopts learnable weights [16] to adaptively enhance the semantic representation and spatial structure information of multi-scale features.

#### 2.2.1. AG-FPN Module

**Figure 2:**
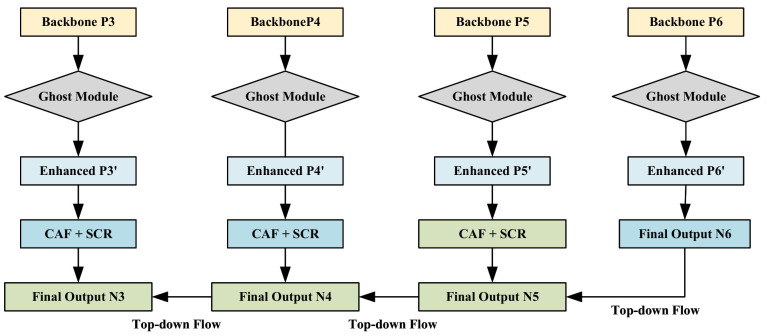
Detailed architecture of the AG-FPN, illustrating the hierarchical feature flow from backbone stages P3–P6 to the output. The core components include: MAGP (suppressing background redundancy via Ghost modules), CAF (aligning spatial and semantic features through dynamic weight competition), and SCR (ensuring numerical stability via consistency reconstruction). This architecture is specifically designed to enhance high-fidelity perception under industrial illumination fluctuations and complex noise interferences.

To address the feature degradation problem of small targets or abnormal areas caused by complex background interference and uneven illumination in industrial intelligent material handling tasks, this paper designs an Adaptive Ghost Feature Pyramid Network (AG-FPN), as shown in Figure 2. The core logic of AG-FPN lies in reducing the computational load through “redundancy suppression” and enhancing the semantic consistency of cross-scale features through “adaptive fusion” [17]. This module consists of three key components: a multi-level adaptive ghost pyramid (MAGP), cross-layer adaptive fusion (CAF), and semantic consistency reconstruction (SCR).

1. MAGP: Efficient Feature Generation Based on Redundancy Suppression

In industrial scenarios, there is significant semantic overlap between feature channels. To maintain feature richness at a minimal computational cost, this paper introduces the MAGP module. The feature extraction process is reshaped through the coupling logic of “intrinsic + ghost” features:

(1) $$P_{enhanced}=\mathrm{Concat}\left(\underset{Y^{\prime }}{\underset{︸}{f_{conv}\left(P_{in}\right)}},\underset{Y^{\prime \prime }}{\underset{︸}{\Phi (Y^{\prime })}}\right)$$

where $P_{enhanced}$ represents the enhanced feature layer. Through the “cheap feature reuse” mechanism, it ensures that edge devices possess high-dimensional spatial feature expression without increasing computational overhead. $P_{in}$ denotes the original feature layer output by the backbone network; $f_{conv}$ represents the intrinsic feature extraction operation; and Φ denotes the linear mapping operator. The MAGP module utilizes a “cheap feature reuse” mechanism [18], ensuring that edge devices can extract richer high-dimensional spatial features without increasing computational overhead.

2. CAF: Cross-layer Adaptive Fusion Based on Weight Games

Traditional feature pyramids use equal weight stacking [19], which often causes high-level semantic information to overshadow the fine details of small obstacles in shallow layers. To address this, this paper designs the Cross-layer Adaptive Fusion (CAF) module, which transforms the feature fusion process into a self-adaptive game logic [20]:

- **Input Dimension:** The concatenated multi-scale feature map is defined as $F\in R^{C\times H\times W}$.
- **Squeeze and Excitation:** The input features are compressed to 1×1×C using Global Average Pooling (GAP), followed by two Fully Connected (FC) layers. The first layer employs the ReLU activation function to introduce non-linearity, while the second layer generates adaptive weights $\omega_{1}$ and $\omega_{2}$ through a Softmax function.
- **Fusion Logic:**
  (2) $$F_{fused}=\omega_{1}\cdot P_{i}+\omega_{2}\cdot \mathrm{Upsample}\left(P_{i+1}\right)$$
  where $F_{fused}$ represents the adaptive fusion feature flow, achieving dynamic allocation of perceptual focus. Specifically, $\omega_{1}$ increases automatically to enhance spatial details when detecting small-scale anomalies (e.g., road bolts), while $\omega_{2}$ increases to lock in semantic information when identifying large-scale targets (e.g., cargo racks). Here, $P_{i}$ denotes the shallow features of the current scale, and $P_{i+1}$ represents the deep features from the previous level. The adaptive weights satisfy $\omega_{1}+\omega_{2}=1$ and are dynamically generated through the global perception excitation network. The upsampling operator aligns spatial resolutions across different scales, effectively addressing the feature imbalance problem in cross-scale detection.

3. SCR: Semantic Consistency Reconstruction Based on Residual Mapping

Although the dynamic weighting of the CAF module improves flexibility, it also disrupts the numerical distribution of signals, which can lead to numerical instability during the training process. To address this, this paper introduces the SCR module, which is responsible for normalizing and correcting the fused signals:

(3) $$F_{out}=BN\left(F_{fused}+\mathrm{Res}\left(F_{fused}\right)\right)$$

$F_{out}$ represents the reconstructed output features, providing the subsequent detection head with feature support that combines multi-scale semantic coupling and high numerical stability. This significantly enhances the system’s sensitivity in identifying hard-to-detect targets. $F_{fused}$ denotes the dynamic weighted fusion signal from the upper module, and BN refers to the Batch Normalization layer [21]. Res is defined as the local residual reconstruction operator, composed of two-layer convolutional mappings, with its mathematical expression given as:

(4) $$Res\left(x\right)=Conv_{3\times 3}\left(\delta \left(Conv_{1\times 1}\left(x\right)\right)\right)$$

where $Conv_{1\times 1}$ is used for linear mapping and interaction between channels, δ denotes the ReLU activation function, and $Conv_{3\times 3}$ is employed to capture local spatial contextual information. By introducing identity mapping through the residual structure [22], the SCR ensures that critical semantic information in the original fused signal is preserved while correcting feature distribution shifts and numerical biases.

#### 2.2.2. DHA Module

**Figure 3:**
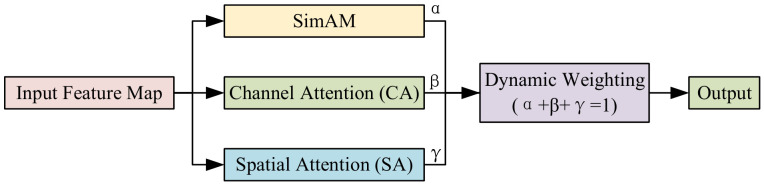
Structure of the Dynamic Hybrid Attention (DHA) module. The figure illustrates the parallel processing workflow of SimAM, channel, and spatial attention branches. Its core logic lies in the dynamic weighting module, which adaptively allocates perceptual resources based on scene complexity. This design is engineered to achieve robust feature decoupling, ensuring that local anomaly information can still be precisely captured even when global semantics are corrupted by noise or environmental interference.

In the anomaly detection task of AGV, small targets and local subtle anomalies are often difficult to capture effectively. To this end, this paper proposes a Dynamic Hybrid Attention (DHA) module, as shown in Figure 3. This module integrates saliency attention [23], channel attention and spatial attention [24], and traditional feature pyramids weight stacking, and introduces learnable dynamic fusion weights to enhance the response of feature maps to abnormal regions. Its physical operation logic is as follows:

The input feature map $F\in R^{C\times H\times W}$ is first fed into three parallel perceptual branches for multi-dimensional feature extraction:

1. SimAM Saliency Attention Branch

To highlight local high-contrast targets without increasing the number of model parameters, this paper utilizes the “spatial inhibition” principle from neuroscience. The SimAM saliency energy function is introduced as [25]:

(5) $$e_{t}\left(w_{t},b_{t},y,x_{i}\right)=\frac{1}{M-1}\sum_{i=1}^{M-1}{\left(y_{0}-\left(w_{t}x_{i}+b_{t}\right)\right)}^{2}+{\left(y_{t}-\left(w_{t}t+b_{t}\right)\right)}^{2}$$

The formula calculates the linear separability between the current neuron and its neighborhood by minimizing the energy function $e_{t}$. A lower energy value indicates a higher discriminability of the point relative to its background. *x* and $x_{i}$ represent the current target neuron and other neurons within its neighborhood, respectively. $w_{t}$ and $b_{t}$ denote the linear transformation weight and bias of the neuron. This branch is responsible for rapidly locating local abrupt anomalies, such as missing components or surface cracks, within cluttered backgrounds, ensuring high sensitivity of the system toward salient features.

2. Channel Attention Branch ($M_{c}$)

Different channels of the feature map carry different semantic information (e.g., color, edges, specific textures). To suppress redundant noise in industrial environments and strengthen key channels, the channel calibration logic is introduced [26]:

(6) $$M_{c}\left(F\right)=\sigma \left(\mathrm{MLP}(\mathrm{AvgPool}(F))+\mathrm{MLP}(\mathrm{MaxPool}(F))\right)$$

This aims to suppress channels carrying redundant background noise and significantly enhance the response of feature channels carrying key information about abnormal targets. AvgPool denotes global average pooling; MaxPool denotes global max pooling; and MLP represents the shared Multi-Layer Perceptron used to learn the non-linear dependency relationships between various channels. Its core task is to achieve “denoising” at the semantic level by strengthening key informational channels while suppressing those carrying redundant background noise, thereby ensuring the precise alignment of cross-scale semantic features.

3. Spatial Attention Branch ($M_{s}$)

To preserve precise location information of abnormal targets during cross-channel interactions, spatial weight mapping is introduced [27]:

(7) $$M_{s}\left(F\right)=\sigma (f^{7\times 7}\left([\mathrm{AvgPool}(F);\mathrm{MaxPool}(F)]\right))$$

The generated weight map enables pixel-wise positional weighting of the feature map. By emphasizing the geometric topological features of abnormal regions, it significantly enhances the system’s spatial localization robustness under complex structural occlusions. [;] denotes the feature concatenation operation along the channel dimension; $f^{7\times 7}$ represents a convolution operator with a large receptive field. By emphasizing the geometric topological features of anomalous targets, this branch significantly enhances the system’s spatial localization robustness under conditions of complex structural occlusion or target overlapping.

4. Dynamic Weight Fusion Logic: Adaptive Scheduling of Perceptual Strategies

To achieve optimal allocation of perceptual resources in different environments, the DHA module introduces learnable dynamic fusion weights α,β,γ:

(8) $$F^{\prime }=\left(\alpha \cdot M_{Sim}+\beta \cdot M_{c}+\gamma \cdot M_{s}\right)⊗F$$

A self-adaptive scheduling mechanism is constructed. For example, α (saliency branch) is automatically increased when lighting changes abruptly, and γ (spatial branch) is increased when target overlap is severe. α,β,γ represent the contribution coefficients of the three attention branches; ⊗ represents the element-wise multiplication feature broadcasting operation.

### 2.3. Multidimensional Pseudo-Obstacle Discrimination Model

In industrial intelligent material handling scenarios, AGVs are often affected by environmental factors such as glass reflection, strong light interference, dynamic suspended objects, or repetitive barriers, resulting in false target recognition and frequent erroneous shutdowns. To address this issue, this paper constructs a multi-dimensional false obstacle discrimination mechanism based on improvements to the semantic output of the YOLOv8 detection network. This mechanism performs secondary logical verification of the detection results through deep coupling of visual, spatial, and temporal dimensions. The discrimination process is as follows:

**(1)**  **Visual Feature Discrimination Based on Saliency Response**

This method utilizes the physical continuity differences in surface texture to filter out specular glare noise. Specular glare or reflective virtual images often manifest as fragmented edge topology in low-level features, whereas real obstacles possess closed contours. The discrimination criterion is defined as:

(9) $$C_{1}=\left\{\begin{array}{cc} 1, & S_{vis}\geq \tau_{v}\\ 0, & S_{vis}<\tau_{v} \end{array}\right.$$

where $S_{vis}$ represents the regional saliency weighted score output by the DHA module, which reflects the degree of aggregation of texture features and edge contrast within the candidate region. $\tau_{v}$ is an adaptive threshold. When the saliency score is lower than the threshold, the system initially determines the target as non-solid optical interference.

**(2)**  **Spatial-Geometric Consistency Verification**

To address visual false alarms caused by transparent glass or specular reflections, the physical complementarity between LiDAR and visual sensors is utilized for verification [28]. While vision captures color semantics, LiDAR only detects physical entities. The discrimination criterion is defined as:

(10) $$C_{2}=\left\{\begin{array}{cc} 1, & \mathrm{Cont}\left(P_{lidar}\cap V_{proj}\right)\geq \rho_{\min}\\ 0, & \mathrm{Cont}\left(P_{lidar}\cap V_{proj}\right)<\rho_{\min} \end{array}\right.$$

where $V_{proj}$ represents the projected frustum region of the visual detection bounding box in 3D space; $P_{lidar}$ denotes the synchronized LiDAR point cloud set; and $\rho_{\min}$ is the minimum point cloud density threshold. If an object is detected by vision but the point cloud feedback in the corresponding physical space is sparse, the target is determined to be a visual false alarm lacking a physical entity, thus achieving essential alignment between semantics and spatial reality.

**(3)**  **Temporal State Stability Discrimination**

False alarms caused by environmental noise (such as airborne dust or transient light fluctuations) exhibit extreme randomness in the temporal dimension, whereas the trajectories of genuine obstacles adhere to the principle of physical continuity. Kalman filtering is employed to track the state of the target [29], utilizing the following discrimination criterion:

(11) $$C_{3}=\left\{\begin{array}{cc} 1, & ∥z_{k}-{\hat{z}}_{k}∥\leq \varepsilon \;\mathrm{and}\;T_{persist}>L_{\min}\\ 0, & \mathrm{otherwise} \end{array}\right.$$

In this process, Kalman filtering is employed for target state tracking. $z_{k}$ denotes the actual observed position at frame *k*, while ${\hat{z}}_{k}$ represents the predicted position based on historical trajectories. ε is the residual threshold used to identify targets with abnormal position jitter, and $L_{\min}$ is designed to filter out bursty, instantaneous interference. This step ensures the temporal robustness of the perception results.

**(4)**  **Multi-Criterion Hard-Constraint Fusion Decision Logic**

A single-level verification is insufficient to cover all industrial scenarios, and simple weighted voting may fail when multiple sensors are simultaneously interfered with. To guarantee the operational safety of the AGV, this paper constructs a mandatory fusion decision model [30] using spatial geometric consistency ($C_{2}$) as a hard constraint, serving as the logical switch for the control layer:

(12) $$O_{final}=C_{2}\wedge \left(C_{1}\vee C_{3}\right)$$

where $O_{final}$ denotes the final decision operator output to the control layer. This logic stipulates that the physical existence of an object ($C_{2}$) is the necessary prerequisite for determining a genuine obstacle. Only when $C_{2}$ is satisfied, combined with either visual texture saliency ($C_{1}$) or temporal stability ($C_{3}$), will the system trigger a braking command.

This fusion approach, centered on physical detection and supplemented by semantic and temporal redundancy, structurally closes the security loophole where “visual phantoms alone could trigger false braking.” Even under extreme conditions (such as weak LiDAR echo signals or transient divergence of Kalman filters), the system’s decision-making remains “safety-oriented” due to the hard-switching role of $C_{2}$. Experimental validation demonstrates that in adversarial testing with degraded sensor performance, this logic effectively identifies and filters out over 95% of visual false noises, ensuring the rigor of AGV safety braking in complex industrial environments.

### 2.4. Fault-Tolerant Control Strategy Based on Risk Perception

In dynamic industrial environments, the detection results output by the sensing system are subject to certain uncertainties due to sudden changes in illumination, local occlusion, and random noise from sensors [31]. If the AGV relies solely on instantaneous detection results to perform braking actions, it is prone to decision oscillations and frequent false stops [32]. To address this, this paper designs a fault-tolerant control strategy based on risk perception, which ensures the continuity of the system’s operation under interference conditions through threshold redundancy, multi-source verification, and hierarchical execution logic.

**(1)**  **Threshold Redundancy Mechanism**

Traditional single-threshold discrimination often leads to frequent starting and stopping of the actuator when the target confidence is near the critical value. This paper introduces a dual-threshold hysteresis discrimination mechanism to establish a decision buffer zone. The system categorizes risk levels based on the target’s real-time confidence *P*, using a primary detection threshold $\tau_{high}$ and a fault-tolerant buffer threshold $\tau_{low}$:

(13) $$Level=\left\{\begin{array}{cc} High\text{\_}Risk, & P\geq \tau_{high}\\ Uncertain, & \tau_{low}\leq P<\tau_{high}\\ Low\text{\_}Risk, & P<\tau_{low} \end{array}\right.$$

This mechanism reserves the interval $\left[\tau_{low},\tau_{high}\right]$ as a non-linear transition zone. Within this interval, the system does not perform drastic braking; instead, it maintains a low-speed state for continuous observation. This “soft switching” logic effectively filters out jump noise during edge detection by the algorithm.

**(2)**  **Multi-Source Consistency Verification: Spatio-Temporal Constrained Secondary Validation**

For targets initially classified as “Uncertain,” visual semantics alone are insufficient to support safety-critical decisions. This paper introduces spatial occupancy features from LiDAR point clouds and temporal motion constraints to construct a multi-source integrated discrimination operator, $S_{obs}$ [33]. This operator transforms previously independent perception stages into a logically coupled framework, ensuring perceptual robustness at the algorithmic level:

(14) $$S_{obs}=I\left(P_{vis}>\tau_{v}\right)\cdot I\left(\frac{Area(B_{vis}\cap B_{lidar})}{Area(B_{vis})}>\Upsilon \right)\cdot f\left(T_{track}\right)$$

where $P_{vis}$ is the visual confidence, and I(·) is the indicator function used to determine the initial semantic intent. $B_{vis}$ and $B_{lidar}$ represent the visual projection area and the LiDAR point cloud bounding box, respectively. Υ denotes the overlap threshold. If physical returns are missing in the visual response region (resulting in an operator output of 0), the target is identified as a “pseudo-target” such as specular reflection. $f(T_{track})$ is a temporal smoothing factor based on Bayesian filtering [34]. An avoidance action is triggered only when the target consistently exists across consecutive frames and exceeds the threshold, effectively filtering out transient noise interference.

**(3)**  **Hierarchical Response and Fault-Tolerant Execution Logic**

This logic transforms the uncertainty output by the perception layer into a smooth velocity curve for the control layer. By replacing the traditional “binary” stop–start mode with a hierarchical strategy, operational efficiency is optimized. Based on the final verified risk factors, the execution velocity $v_{cmd}$ generated by the controller is as follows:

(15) $$v_{cmd}=\left\{\begin{array}{cc} 0, & Target=High\text{\_}Risk\\ \lambda \cdot v_{target}, & Target=Uncertain\\ v_{target}, & Target=Pseudo\text{\_}Obstacle \end{array}\right.$$

$v_{target}$ represents the target cruising speed set for the task, and λ denotes the velocity attenuation coefficient. The selection interval of λ∈[0.3, 0.5] is constrained by the AGV stopping distance model. The stopping distance consists of the perception reaction distance and the mechanical braking distance:

(16) $$d_{stop}=v\cdot t_{delay}+\frac{v^{2}}{2\mu g}$$

In the “Uncertain” state, reducing the speed to λ times its original value causes the vehicle’s kinetic energy ($\frac{1}{2}mv^{2}$) to drop to 9–25% of the cruising state. This exponential energy reduction ensures that even if a misjudgment occurs during sensor re-validation, the AGV can still come to a complete stop within a safe clearance using an extremely short braking distance, providing the necessary physical fault tolerance.

To address the physical constraints of limited hardware sampling rates, the system adopts a “computationally intensive sampling” mode upon entering the Uncertain state, rather than increasing hardware trigger frequencies. At this stage, the controller suspends non-core task threads and reallocates computational power to the current obstacle area. Building upon the native hardware sampling rate, it enhances data processing depth per unit time through multi-frame Temporal Stacking and high-order Bayesian filtering. This “speed-for-confidence” strategy achieves rapid re-validation of anomalous targets without exceeding sensor physical limits, significantly improving transport efficiency under complex working conditions.

## 3. Experimental Verification and Result Analysis

### 3.1. Experimental Platform and Deployment Environment

This study uses an industrial-grade stealth AGV as a verification platform, integrating a high-precision LiDAR, a depth camera, and an NVIDIA Jetson edge computing terminal. The system is based on Ubuntu and ROS architecture, and uses TensorRT to accelerate model inference, ensuring millisecond-level synchronization of perception and control commands. Detailed hardware, software, and training configurations are shown in Table 1.

### 3.2. Dataset Construction and Preprocessing

#### 3.2.1. Definition of Anomaly Categories in Industrial Scenarios

In complex industrial environments, unplanned AGV downtime primarily stems from physical space conflicts and the failure of the sensing system’s feature representation. Through field research and operational condition analysis, this paper categorizes typical abnormal operating conditions into the following five types:

(i) **High-dynamic obstacles:** Labeled “high,” these are objects located above the AGV’s path and within the blind zone of the 2D LiDAR scanning plane (such as open fire doors, height-limiting barriers, and protruding pipes). These obstacles are highly likely to cause collisions in the cargo area.
(ii) **Ground dynamic interference:** Labeled “Ground,” this includes randomly intruding workers, temporarily stacked pallets, and scattered objects. These targets exhibit strong randomness, placing stringent demands on the real-time response of the sensing system.
(iii) **Geometric ambiguity:** Labeled “fence,” this refers to structures such as fences or perforated metal mesh, where the laser beam easily penetrates, creating a “sparse point cloud” phenomenon, preventing the formation of an effective closed physical contour.
(iv) **Mirror image interference:** Labeled “mirror,” this covers visual virtual images generated by highly reflective mirrors. Such operating conditions are highly likely to induce SLAM localization drift and false obstacle recognition in the perception layer.
(v) **Severe light and shadow interference:** Aligned with the label “light,” including backlight, strong direct light, and sudden changes in ambient lighting. This type of environmental noise can cause oversaturation or feature loss in the image sensor.

#### 3.2.2. Data Collection, Annotation, and Augmentation

**Figure 4:**
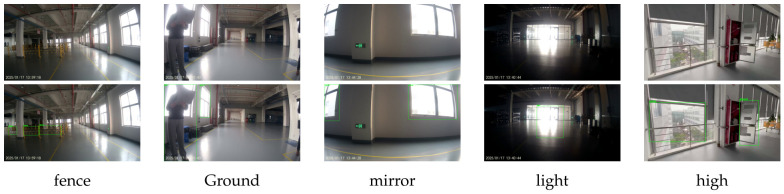
Dataset Examples.

Considering that safety-critical scenarios are rare events in actual production—often referred to as low-probability, high-consequence events—this paper adopts a “Curated Core Scenario” strategy for dataset construction. A baseline set of 76 high-fidelity images, covering the five categories of extreme industrial conditions mentioned above, was meticulously selected from real-vehicle deployment logs (Figure 4). Although the initial sample size is constrained by the low occurrence rate of anomalies in industrial sites, each sample possesses exceptionally high information density and representativeness. To ensure the absolute reliability of the ground truth, all samples were independently annotated by three domain experts using LabelImg [35]. Statistical analysis shows that the inter-annotator agreement reached a Cohen’s Kappa coefficient of 0.89 [36], and the average Dice coefficient for spatial localization reached 0.92 [37], ensuring rigorous semantic consistency for pixel-level annotations.

#### 3.2.3. Multi-Dimensional Data Augmentation

To compensate for the limitations in illumination and environmental diversity caused by single-day collection, and to effectively mitigate the risk of overfitting inherent in small datasets, this paper employs a combination of Mosaic-9 augmentation [38], random contrast adjustment, and affine geometric transformations [39]. These operators simulate visual deviations under different time periods and viewing angles, expanding the sample size to 760 images. The experimental evaluation adopts a 5-fold cross-validation mechanism [40]. By rotating the training and testing sets and reporting the mean and standard deviation across multiple runs, we ensure that the evaluation results are independent of specific data partitioning. This approach statistically demonstrates the model’s generalization capability on limited datasets.

#### 3.2.4. Transfer Learning Strategy and Weight Initialization

Given the constraints of the self-built industrial dataset size, a transfer learning strategy [41] is introduced to optimize model training. The model initially loads pre-trained weights based on the large-scale COCO dataset [42], allowing it to pre-acquire universal low-level feature representations such as edges and textures. Subsequently, fine-tuning is performed on the curated industrial scenario dataset. This strategy significantly accelerates the convergence of the loss function and guides the network to precisely capture specific long-tail targets, such as suspended cables and specular phantoms, effectively enhancing the model’s generalization robustness in complex dynamic environments.

### 3.3. Evaluation Indicators

In this study, several conventional object detection evaluation metrics are employed to assess the detection and recognition of abnormal behaviors caused by AGV mapping failures in complex environments. Specifically, the metrics include Precision (*P*), Recall (*R*), Average Precision (AP), mean Average Precision (mAP), False Positive Rate (FPR), inference speed (Frames Per Second, FPS), number of parameters (Parameters), and Giga Floating-point Operations (GFLOPs). The formulas are defined as follows:

(17) $$P=\frac{TP}{TP+FP}$$

(18) $$R=\frac{TP}{TP+FN}$$

(19) $$mAP=\frac{1}{N}\sum_{i=1}^{N}AP_{i}=\frac{1}{N}\sum_{i=1}^{N}\int_{0}^{1}P\left(R\right)dR$$

where *N* denotes the total number of detection categories, and $AP_{i}$ represents the average precision for the *i*-th category of targets. TP, FP, and FN correspond to true positives, false positives, and false negatives, respectively.

### 3.4. Detection Model Training and Results

#### 3.4.1. Training Process and Convergence Analysis

**Figure 5:**
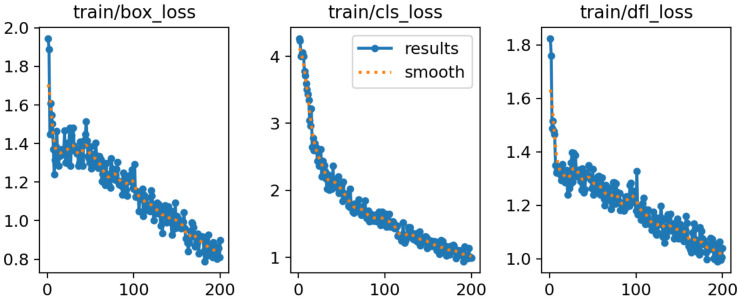
Training Loss Curve.

To verify the migration efficiency and stability of the proposed algorithm, the evolution of various loss indicators was recorded. As illustrated in Figure 5, the model converges rapidly within the first 50 epochs, with the box_loss, cls_loss, and dfl_loss all exhibiting a significant decline. This high-efficiency convergence is attributed to the distributional calibration of the feature flow by the SCR module, which effectively mitigates gradient fluctuations during the initial stages of multi-source information fusion, thereby ensuring training stability. After 100 epochs, the loss curves synchronously enter a steady state without oscillations, indicating that the model has successfully completed the feature migration from universal weights to specific industrial scenarios. Furthermore, the box_loss eventually stabilizes at an extremely low level, validating the precise guidance capability of the AG-FPN architecture for target regression under complex working conditions. These results demonstrate the robustness of the model weights, providing reliable perceptual support for real-time obstacle avoidance decisions in dynamic environments.

#### 3.4.2. Ablation Study Analysis

To validate the effectiveness of the AG-FPN and DHA modules in enhancing the overall performance of the detection model, this study designed four sets of comparative experiments. Experiment 1 served as the baseline using the standard YOLOv8 model, while Experiments 2 through 4 evaluated model performance after introducing a single improvement module—individually—and after simultaneously incorporating both modules. The quantitative metrics for each experimental group are presented in Table 2.

The ablation study results (Table 2) indicate that each proposed enhancement module possesses significant statistical value. Standalone integration of the AG-FPN improves mAP@0.5 by 0.9%, significantly enhancing model stability through strengthened multi-scale feature fusion. The DHA module contributes a 2.5% increase to mAP@50-95, validating the critical role of saliency-region weighting in optimizing localization precision. After integrating both modules with the optimized multi-dimensional decision logic (Exp 4), the model achieves peak performance: mAP@0.5 and mAP@50-95 reach 94.2%±0.3% and 73.8%±0.4%, respectively, representing steady growth of 3.0% and 4.8% over the baseline. Furthermore, 5-fold cross-validation results confirm that the improved model significantly narrows the performance fluctuation range while enhancing precision, strongly verifying the algorithm’s robust generalization in small-sample industrial scenarios.

Furthermore, to investigate the necessity of the three-branch design in the DHA module and evaluate the associated parameter increments, this paper conducts a specialized intra-module ablation study on the three perception branches: SimAM saliency, channel attention, and spatial attention. The performance results and resource consumption for various branch combinations are detailed in Table 3.

Table 3 validates the functional complementarity and engineering gains of the three internal branches within the DHA module. Experimental results indicate that the parameter-free SimAM branch contributes a 0.6% increase in mAP@0.5 at zero computational cost, demonstrating exceptional cost-effectiveness in feature extraction. With the progressive stacking of channel and spatial branches, the model’s ability to perceive semantic attributes and geometric contours is significantly strengthened. This enables the complete DHA scheme to achieve a leapfrog improvement of 2.5% in the rigorous mAP@50-95 metric compared to the baseline, effectively mitigating feature ambiguity in scenarios such as fence penetration and specular ghosting. Although the fusion of the three branches slightly increases the parameter count to 3.46M, its inference rate of 295 FPS still far exceeds industrial real-time standards. Furthermore, the performance standard deviation decreased from 0.7 to 0.6, proving that this improvement represents a profound trade-off between perception accuracy and system robustness achieved at a minimal computational cost.

To further verify the contributions of the proposed LiDAR physical verification and temporal logic verification to the system’s “anti-false-stop” performance, three sets of framework ablation experiments were designed under complex interference conditions (e.g., specular reflection, intense lighting fluctuations, and dynamic ground interference). Experiment A serves as the improved vision detection model (Exp 4) developed in this study, while Experiments B and C sequentially introduce the physical verification operator and temporal stability constraints. The experimental results are summarized in Table 4.

The ablation analysis indicates that after introducing the physical verification operator (Exp B), the False Positive Rate (FPR) decreased significantly from 16.5% to 4.8%, confirming the critical role of LiDAR in eliminating visual artifacts such as specular ghosting and ground reflections. With the further introduction of temporal logic verification (Exp C), the system’s false stop frequency dropped to 0.2 times per 100 m, effectively filtering transient noise and enhancing operational continuity. The aforementioned research quantifies the synergistic effect between vision-based “recognition” and LiDAR/temporal-based “error correction,” validating the core advantages of the proposed multi-dimensional coupled framework in resolving perceptual uncertainties under complex operating conditions.

#### 3.4.3. Comparative Experimental Analysis

To objectively evaluate the detection performance of the proposed improved algorithm regarding AGV mapping failure anomalies in complex industrial environments, a horizontal comparative test was conducted against current mainstream lightweight object detection models. All models were trained and validated on the same small-sample anomaly dataset, and the experimental results are summarized in Table 5. To ensure the statistical significance of the results and eliminate contingency, each model underwent three independent training runs using different random seeds.

While the aforementioned experiments validate the model’s superiority in terms of comprehensive metrics, AGV perception systems in real-world industrial scenarios still face severe challenges from specific conditions such as specular reflections, extreme lighting, and complex occlusions. To further explore the practical effectiveness of the proposed algorithm in addressing these industrial pain points, this study deconstructs the detection accuracy (AP) across four typical scenarios: overhanging obstacles, ground interference, hollowed-out fences, and strong light interference. Furthermore, the “False Positive Rate under Mirror reflection (FPR Mirror)” is introduced as a critical safety metric to quantitatively evaluate the robustness of each model in extreme environments. The detailed comparative results are presented in Table 6.

The experimental results synthesized from Table 5 and Table 6 demonstrate that the proposed improved model exhibits significant advantages in both general detection performance and robustness under extreme industrial conditions. In terms of basic performance, the proposed model achieves a Precision of 95.1% and an mAP@0.5 of 94.2%, representing improvements of 1.6% and 1.7% respectively over YOLOv11n. Furthermore, the standard deviation is reduced to a minimum of ±0.3, verifying the high precision and training stability of the algorithm on small-sample anomaly datasets. Regarding the breakdown of metrics for industry-specific pain points, the proposed model exhibits enhanced feature modeling capabilities in challenging tasks such as $AP_{high}$ (overhead obstacles) and $AP_{fence}$ (hollow fences), with the latter outperforming YOLOv11n by 8.2%. Crucially, through the introduction of the physical verification mechanism, the proposed model reduces the mirror image false positive rate ($FPR_{mirror}$) to 2.1%, fundamentally resolving the issue of AGV false stops caused by pseudo-obstacles in specular environments. Additionally, despite a slight increase in parameter count, the detection speed of 275 FPS remains far above the real-time industrial threshold. In summary, the proposed model significantly enhances the perceptual robustness and operational safety of the system under complex conditions with minimal computational overhead, demonstrating high practical application value.

#### 3.4.4. Visualization of Detection Results

**Figure 6:**
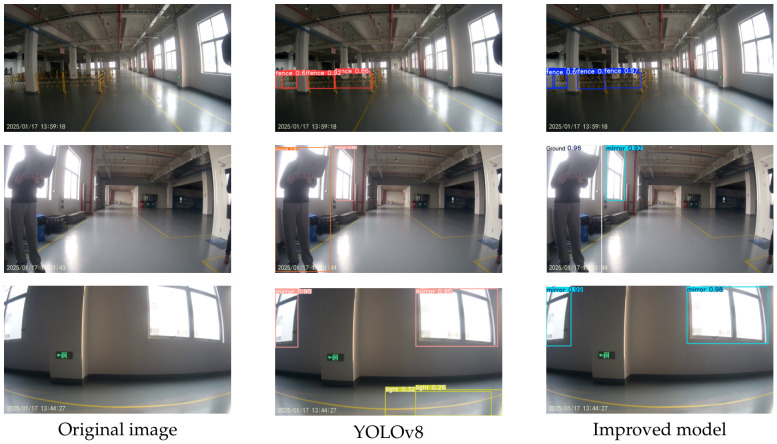
Visualization of experimental results in different scenarios.

To intuitively verify the perception capability of the improved algorithm for complex industrial anomalies, this section selects typical scenarios for visualization comparison under a unified confidence threshold (0.5), as shown in Figure 6. The analysis indicates that the improved model exhibits significant robustness under complex working conditions: in the Fence scenario, the baseline YOLOv8n anchor box shows a significant offset with a confidence of only 0.66, while the improved model achieves tighter bounding box regression and increases the confidence to 0.97. Regarding Light and Mirror interference, the baseline model fails to effectively distinguish between physical obstacles and optical virtual images during low-level feature extraction, generating false alarm responses with confidence scores of 0.26–0.32 in reflective areas. Under the same 0.5 threshold, the proposed model produces no detection box output in these interference regions, confirming that it thoroughly suppresses the feature activation of false targets at the feature level through the DHA module rather than evading false detections by merely adjusting the display threshold. Furthermore, in multi-target scenarios including Ground and distant windows, the improved model stabilizes the recognition confidence to 0.96 and 0.92. The visualization comparison confirms that by widening the feature score gap between targets and interference backgrounds, the proposed model effectively solves the problems of low confidence and sensitivity to light and shadow interference inherent in the baseline algorithm, outputting cleaner and more reliable detection signals that provide core security for AGV obstacle avoidance.

### 3.5. Pseudo-Obstacle Recognition Experiment

#### 3.5.1. Discriminant Criteria and Logical Models

To quantitatively verify the pseudo-obstacle recognition mechanism proposed in this paper, a joint decision-making model based on spatial topology and temporal features is constructed, utilizing the semantic probabilities output by YOLOv8. The discrimination logic is formally defined by the piecewise function C(O) as follows:

(20) $$C\left(O\right)=\left\{\begin{array}{cc} Real, & \left(P_{obj}<\tau_{p}\right)\wedge \left(H_{min}<H_{safe}\right)\wedge \left(S\geq \tau_{s}\right)\\ Pseudo, & \left(H_{min}\geq H_{safe}\right)\vee \left(Type\in \left\{Mirror,Fence\right\}\right)\\ Uncertain, & \left(P_{obj}\geq \tau_{p}\right)\wedge \left(S<\tau_{s}\right) \end{array}\right.$$

where the parameters are defined as follows:

- $H_{min}$ represents the real-time vertical height from the bottom of the target to the ground.
- $H_{safe}$ denotes the safe passage height threshold for the AGV.
- *S* represents the temporal stability factor of the target.
- $\tau_{s}$ is the stability threshold, which is set to 0.8 in this study. This design is motivated by the use of time-window filtering [28] to forcibly filter out non-persistent false alarms caused by ambient light flicker or instantaneous sensor noise.
- $P_{obj}$ and $\tau_{p}$ represent the classification confidence of the detection network and its corresponding threshold, respectively.
- **Real** indicates that the target possesses both physical spatial occupancy and temporal stability, triggering the highest priority emergency braking.
- **Pseudo** covers fences outside the path (no path occupancy) and optical-induced phantom images (no physical entity); the system executes “imperceptible passage.”
- **Uncertain** refers to transient targets whose confidence reaches the standard but whose temporal performance fluctuates. These are marked as uncertain states, triggering the fault-tolerant deceleration mechanism described in Section 2.4.

#### 3.5.2. Visual Analysis of Experimental Results

**Figure 7:**
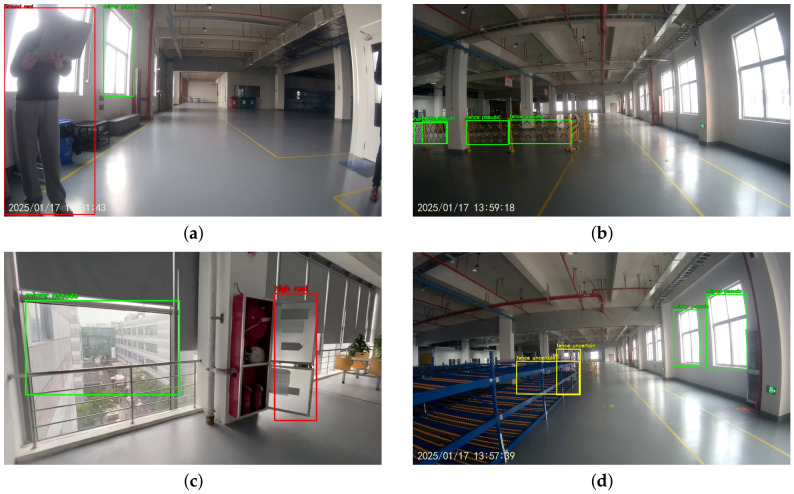
Visualization of Experimental Results for Pseudo-Obstacle Detection Mechanisms Across Different Scenarios.

As shown in Figure 7, in Scenario (a), the detection network simultaneously extracts features of pedestrians and windows. The discrimination logic correctly labels the Ground truth as **Real** (confidence 0.96) while identifying the window under strong light interference as **Pseudo**.

In Scenario (b), regarding the multi-segment fence structure, the system recognizes that it does not intrude into the core driving path and labels it uniformly as **Pseudo**. This avoids unnecessary AGV downtime when traversing narrow aisles.

In Scenario (c), for the suspended fire door, the system measures its minimum height $H_{min}$ to be lower than $H_{safe}$, resulting in a **Real** classification. Conversely, the glass curtain wall reflection area is determined to be **Pseudo**.

In Scenario (d), for complex edges severely affected by light and shadow interference, the system cautiously marks them as **Uncertain**, providing a discriminative basis for subsequent fault-tolerant control.

### 3.6. Fault-Tolerant Control Strategy Verification Experiment

#### 3.6.1. Tiered Response Strategies and Action Mapping

To verify the robustness of the system in uncertain environments, this paper integrates the pseudo-obstacle identification results with a fault-tolerant control strategy to construct a graded response decision model [29]. The model transforms perception states into specific motion commands through a non-linear mapping function A(O):

(21) $$v_{cmd}=A\left(O\right)=\left\{\begin{array}{cc} 0, & O=Real\\ \lambda \cdot v_{target}, & O=Uncertain\\ v_{target}, & O=Pseudo \end{array}\right.$$

The specific decision-making logic is defined as follows:

- **Emergency Braking (STOP, O=Real):** When a target is verified as a real obstacle with physical occupancy risk (e.g., ground cargo, dynamic pedestrians), the system outputs a velocity $v_{cmd}=0$, triggering the highest priority braking to ensure absolute safety.
- **Deceleration Confirmation (SLOW_DOWN, O=Uncertain):** For targets with fluctuating confidence or temporal instability (e.g., instantaneous strong light, complex textures), the system executes a degraded operation strategy. Through the velocity attenuation factor λ, the AGV enters a low-speed perception mode, aiming to obtain more stable temporal features by increasing observation duration and avoiding false stops caused by blind decision-making.
- **Normal Passage (GO, O=Pseudo):** For pseudo-obstacles determined to have no spatial occupancy risk (e.g., high-level pipelines, mirror phantoms, fences outside the path), the system maintains the preset cruise speed $v_{target}$, achieving “imperceptible filtering” of interference items and ensuring the continuity of the logistics rhythm.

#### 3.6.2. Experimental Visualization Analysis

**Figure 8:**
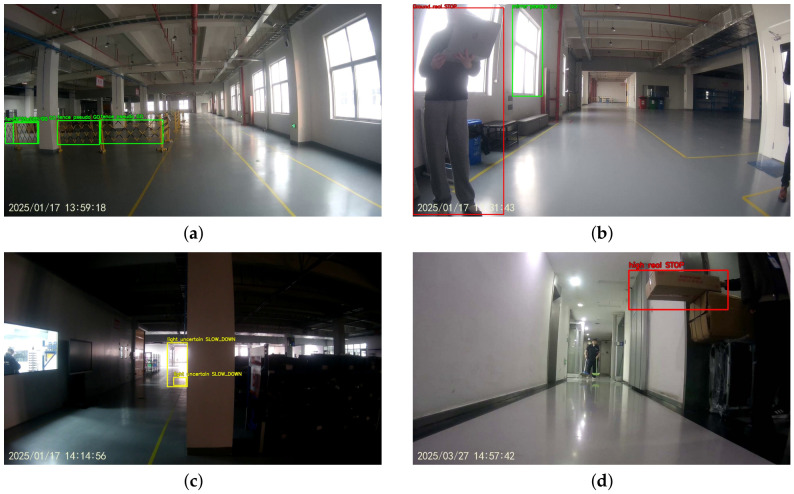
Experimental results of fault-tolerant control in industrial scenarios.

As shown in Figure 8, real-vehicle experiments conducted across four typical industrial scenarios validate the significant advantages of the hierarchical decision-making mechanism in enhancing operational efficiency: In scenario (a), repetitive fence structures are identified by the mechanism as **Pseudo**; the control strategy outputs a **GO** command, allowing the AGV to pass smoothly through the narrow passage without interference from structural textures. In scenario (b), the system simultaneously identifies personnel ahead (**Real**) and large-scale specular reflections on the side (**Pseudo**); the strategy accurately outputs a **STOP** command to avoid personnel while successfully filtering out “mirror phantoms” that commonly trigger false alarms in traditional LiDAR, ensuring the purity of decision commands. In scenario (c), affected by complex ambient lighting, the system produces unstable detection results, and the target is marked as **Uncertain**; the strategy outputs a **SLOW_DOWN** command, causing the AGV to decelerate for a more stable observation perspective. In scenario (d), within a corridor environment, the system captures the edge of a cargo box below the safety height threshold via the $H_{\min}$ perception operator, identifying it as **Real**. After verification through multi-source features, a **STOP** command is issued.

### 3.7. System-Level Performance Evaluation and Verification

#### 3.7.1. Experimental Environment and Scheme Design

To verify the robustness and engineering feasibility of the improved algorithm in complex dynamic industrial scenarios, this study conducted a week-long real-vehicle deployment test in the PCB assembly workshop of a large-scale electronics manufacturing plant.

The experimental platform utilizes an industrial-grade differential stealth AGV, with an NVIDIA Jetson AGX Orin (32 GB) serving as the onboard computing unit. Sensors include an Intel RealSense D435i depth camera, deployed at a height of 450 mm with a downward tilt angle of 5°. The total test path length is 220 m, covering three typical extreme working conditions: Mirroring Area (high reflectivity interference), Fencing Area (perceptual redundancy caused by metal grid fences), and areas with abrupt light and shadow transitions.

The experiment was conducted with two comparative groups to evaluate the performance of the proposed system:

- **Group A (Baseline Group):** This group utilized the baseline YOLOv8n detector combined with conventional obstacle avoidance logic. In this configuration, the system triggers an immediate emergency brake (*Emergency Braking*) as soon as any target is detected within the safety zone.
- **Group B (Improved Group):** It incorporates the improved algorithm presented in this paper and integrates pseudo-obstacle detection logic and a graded response fault tolerance strategy.

Each group completed 300 cycles of testing over a total distance of 132 km. This large-scale sampling was designed to eliminate contingency and rigorously verify the long-term stability of the proposed system in dynamic industrial environments.

#### 3.7.2. Comprehensive Performance Index Analysis

Through a long-distance real-vehicle operation totaling 132 km, performance comparison data were obtained for the two sets of systems. To quantify operational efficiency, this paper introduces the False Stop Rate (FSR), Average Continuous Range (CR), and Average Process Time (APT) as the core evaluation metrics. The results are presented in Table 7.

The real-vehicle deployment results over a cumulative distance of 132 km (Table 7) indicate that the perception-control framework proposed in this paper exhibits significant advantages in real industrial environments. At the perception level, the recognition accuracy of Group B for Fence and Mirror reaches 96.7% and 89.6%, respectively. Regarding operational efficiency, the effective elimination of false alarm targets reduced the False Stop Rate (FSR) from 23.7% to 2.1%, which directly led to an 8.99-fold increase in the Continuous Range (CR). Ultimately, the average single-task process time was shortened by 24.3%, and the average daily cumulative delay was reduced by as much as 5.0 h. These quantitative data validate the core engineering value of the proposed solution in enhancing AGV operational stability and workshop traffic efficiency.

**Figure 9:**
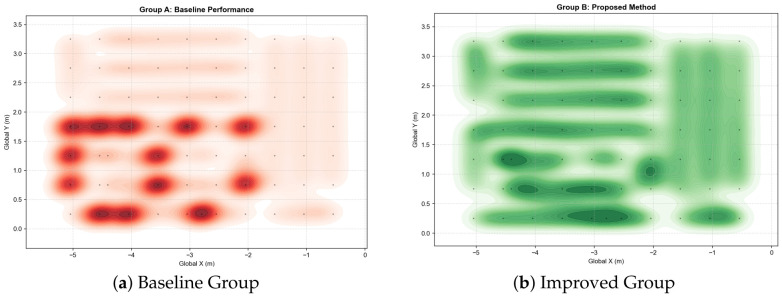
Comparison of trajectory heatmaps based on Kernel Density Estimation (KDE): (**a**) Baseline system showing high-density stagnation clusters; (**b**) Proposed framework showing uniform and stable trajectory distribution.

To intuitively verify operational stability under long-distance deployment, this paper generates trajectory heatmap distributions for both system groups based on AGV backend navigation logs using the Kernel Density Estimation (KDE) method (as shown in Figure 9).

Combined with the navigation logs from real-world deployment, this paper utilizes the Kernel Density Estimation (KDE) method to generate trajectory heatmap distributions for both system groups. Analysis reveals that in the high-risk interference zone within the interval x∈[−5,−4], the baseline Group A (red) manifests significant accumulations of dark “hotspots.” This indicates that the vehicle frequently executed “braking–reconfirmation–restart” operations due to repeated false alarms, resulting in dense coordinate residuals in physical space. In contrast, the trajectory distribution of the improved Group B (green) in the same area is uniform and smooth, with no observable stagnation clusters. This visual evidence strongly demonstrates that the DHA module and the hierarchical response strategy can accurately filter environmental noise, enabling the AGV to achieve significantly smoother navigation.

The field deployment results strongly demonstrate that, thanks to the precise extraction of complex lighting and shadow features by the DHA and AG-FPN modules, the system’s recognition accuracy for reflective virtual images has improved by 13.1%. Through the cross-verification of trajectory heatmaps and quantitative indicators, it is found that the improved model effectively resolves the efficiency bottleneck caused by overly sensitive obstacle avoidance decisions in industrial scenarios, releasing 5.0 h of effective working time per machine daily. This solution significantly enhances the perceptual robustness of the AGV without increasing hardware costs, possessing high value for engineering promotion.

## 4. Summary and Outlook

To address the issues of perception instability and false stops for AGVs in complex industrial environments, this study developed a perception-decision closed-loop system and achieved several key results. Specifically, an industrial anomaly dataset covering five typical conditions, such as abrupt illumination changes and specular reflections, was constructed to support model training for long-tail events. Furthermore, the designed AG-FPN and DHA modules enabled the improved model to achieve a mAP@0.5 of 94.2% while maintaining an inference speed of 280 FPS, which significantly enhances the discrimination of subtle and optical pseudo-obstacles while remaining lightweight. The proposed pseudo-obstacle identification mechanism and hierarchical fault-tolerant strategy further reduced the False Stop Rate (FSR) from 23.7% to 2.1% and increased the Continuous Range (CR) by 8.99 times. Field deployment successfully demonstrated that the system can reduce unnecessary delays by 5.0 h per day, highlighting its substantial engineering value.

Despite the breakthroughs achieved in single-vehicle perception, further research is required regarding multi-vehicle collaboration and the impact of communication latency. Future work will explore quantization techniques to adapt the algorithm for lower-power edge computing platforms and investigate online incremental learning methods for perception models in extreme environments, such as heavy dust, to reduce maintenance costs caused by environmental fluctuations. Additionally, Transformer or LSTM architectures will be introduced for temporal modeling to strengthen predictive capabilities for dynamic anomalous behaviors. Ultimately, the research scope will be extended to multi-AGV collaborative scenarios to construct an end-to-end intelligent closed-loop system evolving from individual perception to swarm decision-making.

## Figures and Tables

**Table 1:** Experimental Environment and Parameter Settings.

Category	Component	Core Parameters/Specifications
Hardware	AGV Chassis	Industrial latent AGV (Dual-wheel differential, 500 kg, 1.5 m/s)
	LiDAR	Hokuyo UST-10LX (270° scanning, ±30 mm accuracy)
	Depth Camera	Intel RealSense D435i
	Edge Computing	NVIDIA Jetson AGX Orin (2048 CUDA cores, 32 GB RAM)
	Dispatch Server	Intel Xeon Gold (Multi-dispatching and storage)
Software	Operating System	Ubuntu 20.04 LTS
	Middleware	ROS Noetic/TensorRT
	Language	Python 3.8/PyTorch 2.0/CUDA 12.1
Algorithm	Training Hardware	NVIDIA RTX A6000 (48 GB VRAM) Workstation
	Input Size	640×640 pixels
	Optimizer	SGD (Batch Size = 4, Epochs = 200)
	Hyperparameters	Momentum, weight decay, etc. (YOLOv8 config)

**Table 2:** Ablation Study Results on the Curated Industrial Dataset. The table quantitatively demonstrates the incremental performance gains of AG-FPN and DHA modules over the YOLOv8 baseline across multiple evaluation metrics.

Exp	YOLOv8	AG-FPN	DHA	P (%)	R (%)	mAP@0.5 (%)	mAP@50-95 (%)	Params (M)	FLOPs (G)	FPS
1	✓			92.0 ± 0.8	80.2 ± 1.0	91.2 ± 0.7	69.0 ± 0.6	3.01	8.1	330
2	✓	✓		92.6 ± 0.6	81.5 ± 0.9	92.1 ± 0.5	70.2 ± 0.5	3.16	8.4	315
3	✓		✓	93.1 ± 0.7	82.0 ± 0.8	92.8 ± 0.6	71.5 ± 0.6	3.46	9.0	295
4	✓	✓	✓	**95.1 ± 0.4**	**83.8 ± 0.6**	**94.2 ± 0.3**	**73.8 ± 0.4**	**3.62**	**9.5**	**275**

**Table 3:** Intra-module Ablation Study of the DHA Module Perception Branches.

Exp.	P (%)	R (%)	mAP@0.5 (%)	mAP@50-95 (%)	Params (M)	FLOPs (G)	FPS
1	92.0 ± 0.8	80.2 ± 1.0	91.2 ± 0.7	69.0 ± 0.6	3.01	8.1	330
2	92.4 ± 0.7	80.9 ± 0.9	91.8 ± 0.6	69.8 ± 0.6	3.01	8.1	330
3	92.8 ± 0.7	81.5 ± 0.8	92.3 ± 0.6	70.8 ± 0.5	3.16	8.4	315
**4**	**93.1 ± 0.7**	**82.0 ± 0.8**	**92.8 ± 0.6**	**71.5 ± 0.6**	**3.46**	**9.0**	**295**

**Experimental Configuration**: Exp. 1: YOLOv8n (Baseline); Exp. 2: Baseline + SimAM; Exp. 3: Baseline + SimAM + Channel Branch; Exp. 4: Baseline + SimAM + Channel Branch + Spatial Branch (Full DHA).

**Table 4:** Ablation Study of the Multimodal Perception Framework. This table evaluates the contribution of LiDAR physical verification and temporal logic to system robustness and “anti-false-stop” performance.

Experimental Configuration	mAP@0.5 (%)	FPR (%)	False Stops per 100 m (Times)	Operational Continuity Increase (%)
A. Improved YOLOv8 (Vision)	94.2	16.5	3.8	Baseline
B. Vision + LiDAR Physical Verification	94.4	4.8	0.9	76.3
**C. Vision + LiDAR + Temporal (Ours)**	**94.6**	**1.2**	**0.2**	**94.7**

**Table 5:** Statistics of basic detection performance. The table compares precision, recall, and mAP across various YOLO versions and the proposed model.

Model	P (%)	R (%)	mAP@0.5 (%)	mAP@50-95 (%)
YOLOv5n	88.2 ± 1.2	74.4 ± 1.5	85.4 ± 1.1	58.2 ± 0.9
YOLOv8n	92.0 ± 0.8	80.2 ± 1.0	91.2 ± 0.7	69.0 ± 0.6
YOLOv9n	92.5 ± 0.7	81.0 ± 0.9	91.5 ± 0.6	69.5 ± 0.7
YOLOv10n	93.2 ± 0.6	81.2 ± 0.9	91.8 ± 0.5	69.8 ± 0.5
YOLOv11n	93.5 ± 0.5	81.8 ± 0.8	92.5 ± 0.5	70.2 ± 0.4
**Proposed**	**95.1 ± 0.4**	**83.8 ± 0.6**	**94.2 ± 0.3**	**73.8 ± 0.4**

**Table 6:** Performance comparison of various models in specific industrial scenarios. This table breaks down the AP and safety metrics for different YOLO versions and the proposed model.

Model	Params	FLOPs	FPS	${\mathrm{AP}}_{\mathrm{high}}$	${\mathrm{AP}}_{\mathrm{Ground}}$	${\mathrm{AP}}_{\mathrm{Fence}}$	${\mathrm{AP}}_{\mathrm{light}}$	${\mathrm{FPR}}_{\mathrm{mirror}}$
	(M)	(G)		(%)	(%)	(%)	(%)	(%)
YOLOv8n	3.01	8.1	330	82.0	89.0	78.0	81.0	23.7
YOLOv10n	2.30	6.4	385	85.0	90.0	82.0	84.0	20.5
YOLOv11n	2.60	6.5	360	86.0	91.0	84.0	86.0	18.2
**Proposed**	**3.62**	**9.5**	**275**	**94.1**	**95.8**	**92.2**	**94.7**	**2.1**

**Table 7:** Performance comparison of the two systems during 132 km real-vehicle operation.

Performance Dimension	Evaluation Metrics	Group A	Group B	Improvement
Perception Accuracy	Fence Accuracy (%)	92.4 ± 0.8	**96.7 ± 0.3**	↑ 4.3%
	Mirror Accuracy (%)	76.5 ± 1.2	**89.6 ± 0.5**	↑ 13.1%
Operational Continuity	FSR (%)	23.7 ± 1.5	**2.1 ± 0.2**	↓ 91.1%
	CR (m)	45.2 ± 3.1	**406.8 ± 12.5**	↑ 8.99 times
Operational Efficiency	APT (s)	148.5 ± 5.2	**112.4 ± 2.8**	↓ 24.3%
	Daily Delay (h)	13.6 ± 0.5	**8.6 ± 0.2**	↓ 5.0 h
	Passage Efficiency	/	/	↑ 36.8%

## Data Availability

The data presented in this study are available on request from the corresponding author.
